# Intramuscular Vaccination in Adults with Therapeutic Anticoagulation in the Era of COVID-19 Vaccines Outbreak: A Practical Review

**DOI:** 10.1055/s-0041-1729627

**Published:** 2021-05-25

**Authors:** Germain Perrin, Christine Le Beller, Luc Darnige, Lina Khider, David M. Smadja, Agnès Lillo-Le Louet, Benjamin Planquette, David Lebeaux, Olivier Sanchez, Brigitte Sabatier, Tristan Mirault, Nicolas Gendron

**Affiliations:** 1 Département de Pharmacie, Assistance Publique Hôpitaux de Paris, Centre-Université de Paris (APHP-CUP), Information Sciences to support Personalized Medicine, Université de Paris, INSERM, Paris, France; 2 Département de Pharmacovigilance, Assistance Publique Hôpitaux de Paris.Centre-Université de Paris (APHP-CUP), Université de Paris, Innovative Therapies in Haemostasis, INSERM, Paris, France; 3 Hematology department and Biosurgical Research Lab (Carpentier Foundation), Assistance Publique Hôpitaux de Paris.Centre-Université de Paris (APHP-CUP), Université de Paris, Innovative Therapies in Haemostasis, INSERM, Paris, France; 4 Vascular Medicine Department and Biosurgical Research Lab (Carpentier Foundation), Assistance Publique Hôpitaux de Paris-Centre (APHP-CUP), Université de Paris, PARCC U970 INSERM Paris, France; 5 F-CRIN INNOVTE, Saint-Étienne, France; 6 Respiratory Medicine Department and Biosurgical Research Lab (Carpentier Foundation), Assistance Publique - Hôpitaux de Paris-Centre (APHP-CUP), Université de Paris, Innovative Therapies in Haemostasis, INSERM, Paris, France; 7 Infectious Disease Department, Assistance Publique Hôpitaux de Paris, Centre-Université de Paris (APHP-CUP), Université de Paris, Paris, France

## Background

The coronavirus disease 2019 (COVID-19) outbreak has urged governments worldwide to implement measures against the spread of severe acute respiratory syndrome coronavirus 2 (SARS-CoV-2). Even so, pandemic is still poorly controlled, and mass vaccination programs are urgently required to lower SARS-CoV-2 circulation and the pressure on health systems.

According to the World Health Organization, 10 vaccines have been approved, and 64 are currently under development at the date of January 20, 2021. 1 Most of them are administered intramuscularly (IM), because this route of administration is associated with a higher immunogenicity and an improved local tolerance, as compared with subcutaneous (SC) injections. 2 In the perspective of mass vaccination campaigns, concerns emerge for the management of IM injections for patients with therapeutic anticoagulation. Indeed, IM injections are generally discouraged in patients receiving anticoagulant, based on the risk of bleeding and muscle hematomas. 3 Burden of anticoagulant utilization is large in the general population. In France, for instance, 4 million patients had at least one anticoagulant drug reimbursement in 2019 (9% of French population), notably for chronic conditions such as atrial fibrillation. 4

We aimed to discuss the bleeding risk associated with IM injections, and particularly vaccines, in patients with therapeutic anticoagulation, and to propose guidance in clinical practice, in the context of ongoing mass vaccination campaigns.

## What Is the Bleeding Risk Associated with IM Vaccination in Patients with Therapeutic Anticoagulation?

Although avoiding IM injection in patients under anticoagulant is a widespread clinical practice, high-quality evidence supporting this precaution is sparse, and studies are generally focused on IM influenza vaccination in patients under vitamin K antagonist (VKA).

An observational study evaluated this risk in 19 patients (median age: 65 years, 63% men) under therapeutic anticoagulation and/or antiplatelet therapy, who received IM vaccines with a firm pressure, without rubbing, during 2 minutes (min). 5 No bleeding complications were reported. Raj et al. evaluated the safety of IM influenza vaccination in 41 adult patients taking VKA. 6 No significant change in arm circumference, local complication, or bleeding episode was observed between baseline and 3, 7, and 14 days after injection. Delafuente et al performed a single-blind, randomized clinical trial (RCT) to compare the safety of administrating influenza vaccination in the deltoid muscle in 36 men aged over 60 years treated with warfarine. 7 No difference in the rate of local complications between IM and SC was observed. Casajuana et al performed an RCT, enrolling 229 adult patients on VKA, to test safety of IM versus SC influenza vaccine. 8 Subjects had no history of major bleeding, and an international normalized ratio (INR) lower than 4 at the time of enrolment or in the last 2 months. The SC group received one dose influenza vaccine through a preloaded syringe with a needle of 16 mm (⅝ inches) in length and 0.5 mm (25-gauge) in diameter in the deltoid region. The IM group received the same vaccine using the same needle and syringe combination in the same place by IM route. No significant changes in the arm circumference at the site of injection at 24 hours or occurrence of major bleeding were observed. Local reactions, mainly erythema, plaque, and pruritis, were more frequent in the SC group. Hematoma occurred in three patients and one patient in the SC and the IM group respectively, both with basal INR between 2 and 3.

In line with the growing number of patients treated with direct oral anticoagulants (DOACs) instead of VKA, generalizing these conclusions to patients receiving IM vaccine is hazardous. In a study based on the prospective Dresden NOAC registry, 9 authors analyzed peri-interventional safety data from 2,179 patients under DOACs. They classified IM injections as minor procedures (procedures associated with little tissue trauma, but relevant bleeding risk, along with transluminal cardiac, arterial, and venous interventions, pacemaker-related surgery, pleural and ascites punctures, cataract surgery, arthroscopy, endoscopy, laparoscopy, organ biopsies, dental extraction, and hernia repair). Twenty-nine bleeding events were observed after 641 minor procedures (4.5%, 95% confidence interval [CI]: 3.1–6.4). Among them, three were classified as major bleedings (0.5%, 95% CI: 0.0–1.4); none of them were reported after IM injection. Authors concluded that for nonmajor invasive procedures, rate of complication was low and fatal complications seem to be very rare. However, the proportion of patients who underwent IM injection without holding DOACs was not reported. Therefore, continuing DOACs or doing a short-term interruption, without heparin bridging therapy, were two safe options in minor invasive procedures.

The absence of safety concern about a potential risk of bleeding associated with IM vaccination in patients treated with anticoagulants was confirmed by a request performed (January 1, 2021) in the French national pharmacovigilance database.

Altogether, literature seems to indicate an acceptable safety profile of performing a unique IM administration in patients with anticoagulant, especially if general principles of precaution are respected. However, due to the small sample size of these studies, local adverse events could have been missed if their frequency was low.

## What Are the Guidelines?

Several national guidelines described the practical aspects of vaccination, including the issue of IM vaccination in patients under anticoagulants.

Concerning the bleeding risk associated with IM vaccination, the Public Health England addresses the specific case of administering COVID-19 vaccine to individuals taking anticoagulants. 10 This guideline stated that individuals on stable anticoagulation therapy, including individuals on VKA who are up-to-date with their scheduled INR testing and whose latest INR was below the upper threshold of their therapeutic range, can receive IM vaccination. If there is any doubt, a consultation with the clinician responsible for prescribing or monitoring the individual's anticoagulant therapy is recommended. This bleeding risk is also mentioned in other national guidelines. 2 11 Additionally, the Advisory Committee on Immunization Practices recommended that vaccination should be scheduled prior to the use of anticoagulant when possible. 3

For the choice of the route and site of injection, the French practical guide for vaccination stated in 2012 that in thrombocytopenic or hemophilia patients, or patients with anticoagulants, it is recommended to inject the vaccine SC since IM injection can cause bleedings. 11 Nevertheless, the German Standing Committee on Vaccination recommendations (STIKO) at the Robert Koch Institute highlighted the risk of administrating adsorbate vaccines into the SC fatty tissue, since this can be associated with painful inflammation and the formation of granulomas or cysts. STIKO recommendations also raised concerns about the success of vaccination when injected into fatty tissue. The recommended site of IM injection is the deltoid muscle. 2

The application of a local firm pressure, without rubbing, is recommended during 2 to 5 minutes after injection, for both IM and SC routes 3 11 during vaccination procedure in patients with bleeding disorder or under anticoagulant. The use of a fine needle (23-gauge or smaller caliber) is also recommended to further lower the bleeding risk. 3 11 The patient or family should be given information on the risk for hematoma from the injection.

Medical information, provided by vaccines and anticoagulants products monographs inconsistently report the bleeding risk of IM injection in patients with bleeding disorders or with anticoagulant. Product monographs of vaccines mention, when approved, the possible switch from the IM to the SC route, based on validation studies confirming a sufficient immunogenicity and local tolerance with the SC injection ( Table 1 ). Concerning anticoagulants, the warning for IM administration is present in the product monograph of warfarin, but not in that of apixaban, dabigatran, edoxaban, or rivaroxaban.

**Table 1:** Examples of information provided in the product monographs of vaccines

	Usage warning for patients with anticoagulation	Is SC injection authorized in the product monograph?
Pneumococcal vaccine		
Pneumovax	No	Yes
Prevenar 13	Yes	Yes
Influenza vaccine		
Vaxigrip Tetra	Yes	Yes
Influvac Tetra	Yes	Yes
Diphtheria, tetanus, and whooping cough vaccine		
Revaxis	Yes	Yes
Varicella-zoster virus		
Zostavax	Yes	Yes
COVID-19 vaccines		
Moderna + National Institute of Allergy and Infectious Diseases RNA-based vaccine	Yes, deltoid muscle, needle size not specified	SC not authorized
Pfizer/BioNTech + Fosun Pharma RNA-based vaccine	Yes, deltoid muscle, 21-Gauge or narrower	SC not authorized
AstraZeneca + University of Oxford viral vector vaccine	Yes, deltoid muscle, needle size not specified	No specified and no adsorbate in the vaccine formulation
Sinovac Research and Development Co., Ltd	Yes, exclusion criteria in phase III trial (NCT04456595) deltoid muscle, needle size not specified	No product monograph available, presence of aluminum hydroxide
Sinopharm + China National Biotec Group Co + Wuhan Institute of Biological Products	Yes, exclusion criteria in phase III trial (ChiCTR2000034780), injection site not specified, needle size not specified	No product monograph available, presence of aluminum hydroxide
CanSino Biological Inc./Beijing Institute of Biotechnology	No, not an exclusion criterion in phase III trial (NCT04691908), deltoid muscle, needle size not specified	No product monograph available
Gamaleya Research Institute; Health Ministry of the Russian Federation	No, not an exclusion criterion in phase III trial (NCT04530396), Injection site not specified, needle size not specified	No product monograph available
Janssen Pharmaceutical	No, not an exclusion criterion in phase III trial (NCT04505722), injection site not specified, needle size not specified	No product monograph available
Novavax	No, not an exclusion criterion in phase III trial (NCT04611802), deltoid muscle, needle size not specified	No product monograph available
Anhui Zhifei Longcom Biopharmaceutical + Institute of Microbiology, Chinese Academy of Sciences	Yes, exclusion criteria in phase II trial (NCT04466085), deltoid muscle, needle size not specified	No product monograph available
CureVac AG	Yes, exclusion criteria in phase III trial (NCT04674189), deltoid muscle, needle size not specified	No product monograph available
Institute of Medical Biology + Chinese Academy of Medical Sciences	Yes, exclusion criteria in the phase III trial (NCT04659239), injection site not specified, needle size not specified	No product monograph available
Research Institute for Biological Safety Problems, Rep of Kazakhstan	No, not an exclusion criterion in phase III trial (NCT04691908), injection site not specified, needle size not specified	No product monograph available
Bharat Biotech International Limited	No, not an exclusion criterion in phase III trial (NCT04641481), injection site not specified, needle size not specified	No product monograph available, presence of aluminum hydroxide

Abbreviations: COVID-19, coronavirus disease 2019; RNA, ribonucleic acid; SC, subcutaneous.

## Practical Considerations before Vaccinating a Patient Taking Therapeutic anticoagulation

**Fig. 1:**
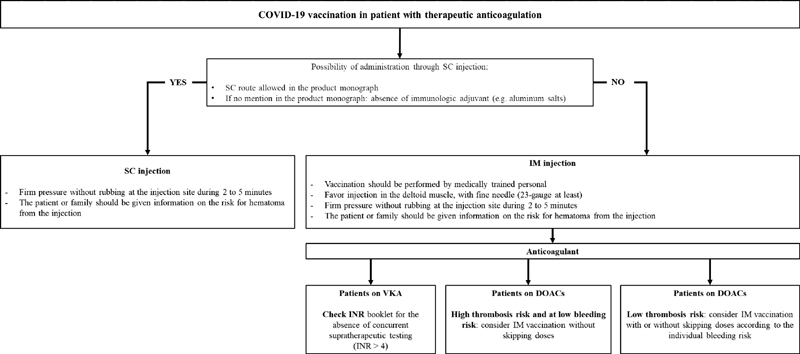
Management of intramuscular vaccination in patients with therapeutic anticoagulation: example of COVID-19 vaccination. COVID-19, coronavirus disease 2019; DOAC, direct oral anticoagulant; IM, intramuscular; SC, subcutaneous; VKA, vitamin K antagonist.

Practical aspects should be considered before vaccinating a subject with therapeutic anticoagulant by IM route (see Fig. 1 for example of COVID-19 vaccination):

1. If therapeutic anticoagulation should end soon, physician may delay the IM vaccination after.
2. If the vaccine monograph mentions SC injection as an alternative to IM injection, physician should prefer SC route over IM route.
3. If the vaccine monograph does not mention alternative to IM injection, SC route may be considered after checking for the absence of adjuvants in the vaccine. Indeed, adjuvants, particularly aluminum salts, can cause pain, cysts, and granuloma at the site of injection. 2 If postvaccination serologic testing is available, efficacy of the vaccination should be assessed as immunogenicity can be compromised using this route.
4. In the absence of SC route as an alternative to IM injection and for vaccines with adjuvants, clinician should use IM route.
  VKA treated-patients, with stable anticoagulation therapy, who are up-to-date with their scheduled INR testing and whose latest INR was below the upper threshold of their therapeutic range and in all cases below 4, can receive IM vaccination. If there is any doubt, a consultation with the clinician responsible for prescribing or monitoring the individual's anticoagulant therapy is recommended.
For DOAC treated-patients, two strategies may be considered, as mentioned in a review 12 by Spyropoulos et al. for minimal bleeding risk procedures: either to not interrupt or to consider interrupting on the day of the procedure the DOAC therapy. The skipping strategy may apply to DOACs because of their favorable pharmacokinetics properties with shorter half-life and shorter C _max_ compared with VKA. 9 For example, the French Working Group on Perioperative Hemostasis proposed not to administer DOAC the evening before and the morning of procedures at low bleeding risk. 13 This could then be applied to patients at low thrombosis risk, especially those at high bleeding risk (e.g., older people, presence of renal failure, liver failure, antiplatelet therapy, bleeding history, and uncontrolled hypertension). For patients at high thrombosis risk and low bleeding risk, IM vaccination may be considered without skipping a dose.
5. In all cases, vaccination should be performed by medically trained personal. For IM route, the injection should be performed in the deltoid muscle, with a fine needle (23-gauge at least). A firm pressure, without rubbing, at the injection site should be maintained 2 to 5 minutes after SC or IM injection. The patient or family should be given information on the risk for hematoma from the injection.

## Conclusion

Even though available data suggest a limited risk for bleeding complications after IM injection in patients under therapeutic anticoagulation, a reminder of global precautions seems helpful in the era of COVID-19 vaccines outbreak, where vaccinations could be largely performed outside the general practitioner's office. A careful risk to benefit evaluation should be performed before vaccinating a patient under anticoagulant by IM route. We encourage people to have vaccinations in particular and they should not be excluded because of therapeutic anticoagulation or bleeding risk.

## References

[1] World Health Organization (WHO) Draft landscape of COVID-19 candidate vaccines [Internet][cited 2020 Dec 16]. Accessed March 25, 2021 from:https://www.who.int/publications/m/item/draft-landscape-of-covid-19-candidate-vaccines

[2] RKI - STIKO Recommendations - STIKO vaccination recommendations 2017/18 [Internet][cited 2020 Dec 12]. Accessed March 25, 2021 from:https://www.rki.de/EN/Content/infections/Vaccination/recommandations/34_2017_engl.html

[3] ACIP General Best Practice Guidelines for Immunization | Recommendations | CDC [Internet]2020 [cited 2020 Dec 12]. Accessed March 25, 2021 from:https://www.cdc.gov/vaccines/hcp/acip-recs/general-recs/index.html

[4] Open Medic: base complète sur les dépenses de médicaments interrégimes - data.gouv.fr [Internet][cited 2019 Sep 2]. Available from:/fr/datasets/open-medic-base-complete-sur-les-depenses-de-medicaments-interregimes

[5] van Aalsburg R van Genderen P JJ Vaccination in patients on anticoagulants Travel Med Infect Dis 2011 9 06 310 311 22018976 10.1016/j.tmaid.2011.09.001

[6] Raj G Kumar R McKinney W P Safety of intramuscular influenza immunization among patients receiving long-term warfarin anticoagulation therapy Arch Intern Med 1995 155 14 1529 1531 7605155

[7] Delafuente J C Davis J A Meuleman J R Jones R A Influenza vaccination and warfarin anticoagulation: a comparison of subcutaneous and intramuscular routes of administration in elderly men Pharmacotherapy 1998 18 03 631 636 9620115

[8] Casajuana J Iglesias B Fàbregas M Safety of intramuscular influenza vaccine in patients receiving oral anticoagulation therapy: a single blinded multi-centre randomized controlled clinical trial BMC Blood Disord 2008 8 1 18507871 10.1186/1471-2326-8-1 PMC2423363

[9] Beyer-Westendorf J Gelbricht V Förster K Peri-interventional management of novel oral anticoagulants in daily care: results from the prospective Dresden NOAC registry Eur Heart J 2014 35 28 1888 1896 24394381 10.1093/eurheartj/eht557

[10] Public Health England COVID-19 vaccination programme Information for healthcare practitioners[internet], [cited 2020 Dec 12] from:https://www.gov.uk/government/publications/covid-19-vaccination-programme-guidance-for-healthcare-practitioners

[11] Direction générale de la Santé, Comité technique des vaccinations. Guide des vaccinations Édition 2012 Saint-Denis Inpes, coll. Varia 2012 488. Available from:https://solidarites-sante.gouv.fr/IMG/pdf/Guide_des_vaccinations_edition_2012.pdf

[12] Spyropoulos A C Al-Badri A Sherwood M W Douketis J D Periprocedural management of patients receiving a vitamin K antagonist or a direct oral anticoagulant requiring an elective procedure or surgery J Thromb Haemost 2016 14 05 875 885 26988871 10.1111/jth.13305

[13] French Working Group on Perioperative Hemostasis (GIHP) Albaladejo P Bonhomme F Blais N Management of direct oral anticoagulants in patients undergoing elective surgeries and invasive procedures: Updated guidelines from the French Working Group on Perioperative Hemostasis (GIHP) - September 2015 Anaesth Crit Care Pain Med 2017 36 01 73 76 27659969 10.1016/j.accpm.2016.09.002

